# OER catalytic performance of a composite catalyst comprising multi-layer thin flake Co_3_O_4_ and PPy nanofibers†

**DOI:** 10.1039/d3ra05936g

**Published:** 2023-10-31

**Authors:** Honglin Ai, Liquan Fan, Yuwei Wang, Ziteng Wang, Haiming Zhang, Juan Zhao, Meiye Jiao, Boyu Lv, Xianxin Han

**Affiliations:** a College of Materials Science and Engineering, Heilongjiang Provincial Key Laboratory of Polymeric Composite Materials, Qiqihar University No. 42, Wenhua Street Qiqihar 161006 PR China Liquan_Fan@163.com wyw032378@163.com

## Abstract

The oxygen evolution reaction (OER) plays a crucial role in energy conversion and storage processes, highlighting the significance of searching for efficient and stable OER catalysts. In this study, we have developed a composite catalyst, PPy@Co_3_O_4_, with outstanding catalytic performance for the OER. The catalyst was constructed by integrating multi-layer thin flake Co_3_O_4_ with attached PPy nanofibers, utilizing the rich active sites of Co_3_O_4_ and the flexibility and tunability of PPy nanofibers to optimize the catalyst structure. Through comprehensive characterization and performance evaluation, our results demonstrate that the PPy@Co_3_O_4_ (0.1 : 1) catalyst exhibits remarkable OER catalytic activity and stability. This research provides new strategies and insights for the development of efficient and stable OER catalysts, holding promising prospects for energy conversion and storage applications in relevant fields.

## Abstract 2

Through synergy between multi-layer Co_3_O_4_ and PPy nanofibers, our catalyst optimizes structure, harnessing rich Co_3_O_4_ sites and PPy flexibility for enhanced OER catalytic performance.

## Graphical abstract

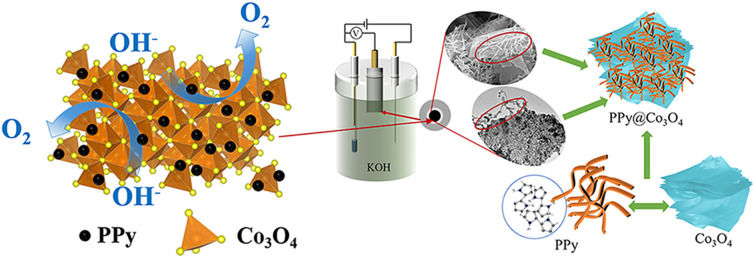

## Introduction

With the rapid development of the fossil fuel industry, such as natural gas, petroleum, and methane, the world is facing severe energy shortages and environmental pollution crises. To propel human society towards further advancement and address challenges of resource scarcity and environmental pollution, the exploration of renewable and recyclable clean energy has become a key research area in this century.^1,2^ Among them, electrocatalytic water splitting is widely regarded as a suitable alternative, primarily involving the oxygen evolution reaction (OER) and hydrogen evolution reaction (HER).^3,4^ Achieving outstanding OER activity requires metals to provide good electron conductivity, thereby enlarging the surface area of active centers for OER catalysis and protecting metals from oxidation through layered structures, resulting in superior OER performance.^5,6^ Currently, noble metal catalysts such as RuO_2_/IrO_2_ are widely employed in OER testing or used as reference materials for comparison due to their exceptional OER catalytic activity. However, their high cost and limited availability restrict further research and application. Therefore, there is an urgent need to develop cost-effective, highly stable, and electrochemically active electrocatalysts.^7,8^

As a transition metal, metal cobalt and its oxide Co_3_O_4_ have attracted significant attention due to their excellent oxygen evolution reaction (OER) performance.^9^ Various catalysts with diverse structures and exposed crystal facets, such as particulate, spherical, bulk, bouquet-like, and network structures, can be obtained by employing different preparation methods, which profoundly influence the catalytic activity in different fields.^10–12^ However, despite certain progress being made, challenges still exist in current research, necessitating further exploration and optimization of the performance of Co_3_O_4_ catalysts to achieve more efficient and stable OER reactions.

To further enhance the OER catalytic performance of Co_3_O_4_, this study introduces a conductive polymer material, polypyrrole (PPy). As a commonly studied and utilized conducting polymer with alternating single-double bond conjugation, PPy possesses a C–N five-membered heterocyclic conjugated structure and finds extensive applications in electrochemical fields such as supercapacitors, oxygen reduction reactions (ORR), and oxygen evolution reactions (OER).^13–15^ The incorporation of PPy enhances the conductivity of the nanocomposite, effectively facilitating electron transfer in the OER process and thus improving the electrocatalytic performance of OER.^16^ In composite materials, the morphology of Co_3_O_4_ and its combination with PPy play a crucial role in their performance in the electrocatalysis field. For instance, a hierarchical Co_3_O_4_@PPy core–shell nanowire can be prepared by uniformly coating an amorphous PPy layer on the surface of Co_3_O_4_ nanowires *via* a simple hydrothermal method, and this composite material has been employed as an electrode material for supercapacitors.^17,18^ In this study, a hydrothermal method combined with the *in situ* polymerization technique using polyvinylpyrrolidone (PVP) as a soft template was employed to synthesize multi-layered flake-like Co_3_O_4_, followed by the preparation of PPy@Co_3_O_4_ composite material. The catalytic performance of PPy@Co_3_O_4_ composite material in the OER reaction was investigated, aiming to provide new insights and theoretical guidance for the development of efficient and stable electrocatalysts.

## Experimental section

### Materials

Cobalt(ii) nitrate hexahydrate (Co(NO_3_)_3_·6H_2_O) was purchased from Xilong Chemical Co., Ltd. Pyrrole was obtained from Macklin Reagent Company. Ammonium persulfate was purchased from Tianjin Kaitong Chemical Reagent Co., Ltd. Urea ((NH_2_)_2_CO) and sodium dodecylbenzenesulfonate (SDBS) were obtained from Tianjin Kemiou Chemical Reagent Co., Ltd. Polyvinylpyrrolidone (PVP) was purchased from Tianjin Bodi Chemical Co., Ltd. All chemicals used were of analytical grade and did not require further purification. The ultrapure water used in this experiment had a resistivity of 18.2 MΩ cm.

### Preparation methods

Co(NO_3_)_3_·6H_2_O and (NH_2_)_2_CO were mixed in a molar ratio of 1 : 5 in 70 mL of ultrapure water. While stirring, an appropriate amount of PVP was gradually added to the mixture solution and stirred overnight until complete dissolution and mixing. The solution was transferred to a 100 mL polytetrafluoroethylene hydrothermal reaction vessel and reacted at 120 °C for 6 h. After the reaction, the reaction vessel was cooled to room temperature, and the precipitate was centrifuged. The centrifuged sample was washed three times with alternating ultrapure water and anhydrous ethanol solution. The sample was then placed in a hot air oven and dried at 80 °C for 6 h. After drying, the sample was removed and placed in a high-temperature furnace. The temperature was raised to 400 °C at a heating rate of 5 °C min^−1^ in air and calcined for 4 hours, resulting in black Co_3_O_4_ powder.

Approximately 0.3 g of the prepared black Co_3_O_4_ powder was taken and added to 50 mL of ultrapure water. Then, 10 mg of SDBS was added, and the mixture was stirred overnight for 24 h. The solution was transferred to a water bath at 0–5 °C and stirred in an ice bath. Then, 30 mg of pyrrole monomer and 0.5 mL of 1 mol L^−1^ hydrochloric acid were added. After stirring for 1 hour, 2 mL of 0.1 mol L^−1^ ammonium persulfate ((NH_4_)_2_S_2_O_8_) was added dropwise. After stirring for 2 h, the mixture was centrifuged at low speed, and the resulting sample was washed three times with alternating ultrapure water and anhydrous ethanol. The sample was then placed in a hot air oven set at 70 °C and dried for 8 hours to obtain the PPy@Co_3_O_4_ composite with a mass ratio of 0.1 : 1, denoted as PPy@Co_3_O_4_ (0.1 : 1). For comparison experiments, other PPy@Co_3_O_4_ composite materials with mass ratios of 0.02 : 1, 0.05 : 1, and 0.2 : 1 were prepared by adjusting the amount of pyrrole monomer under the same conditions. These samples were denoted as PPy@Co_3_O_4_ (0.02 : 1), PPy@Co_3_O_4_ (0.05 : 1), and PPy@Co_3_O_4_ (0.2 : 1), respectively. The preparation of pure PPy was conducted under the same conditions without the addition of Co_3_O_4_.

### Characterization

To analyze the crystal structure and phase composition of the prepared samples, we employed the Smart Lab X-ray diffractometer (XRD) from Rigaku Corporation, Japan. The microscopic morphology of the materials was observed using the S-3400 scanning electron microscope (SEM) from Hitachi Ltd., Japan. Elemental scanning analysis was carried out with an energy dispersive X-ray spectroscope (EDS). Additionally, we utilized the H-7650 transmission electron microscope (TEM) from Hitachi Ltd. to observe the samples. For the observation of PPy in the PPy@Co_3_O_4_ composite, Fourier-transform infrared spectroscopy (FT-IR) analysis was performed using the Spec drum infrared spectrometer from PerkinElmer, USA. The chemical environment of elements in the PPy@Co_3_O_4_ composite was analyzed using the ESCALAB 250Xi X-ray photoelectron spectrometer (XPS) from Thermo Fisher Scientific, USA. The Brunauer–Emmett–Teller (BET) method was employed to determine the specific surface area using a Hitachi Regulus 8100 instrument. The Barrett–Joyner–Halenda (BJH) method was utilized to assess the pore size distribution.

### Electrochemical testing

Electrochemical performance testing was conducted using a Shanghai CHI 760E electrochemical workstation in a 0.1 M KOH electrolyte solution. A three-electrode system was employed, with a catalyst-modified glassy carbon electrode as the working electrode, an Ag/AgCl electrode as the reference electrode, and a platinum wire electrode as the counter electrode. Prior to each test, the glassy carbon electrode was carefully polished using aluminum oxide powder with particle sizes of 0.1, 0.3, and 0.05 mm, followed by rinsing with deionized water. Electrochemical impedance spectroscopy (EIS) measurements were performed over a frequency range of 0.1 to 10^6^ Hz with an amplitude of 5 mV. The double-layer capacitance (*C*_dl_) is estimated from the cyclic voltammetry (CV) curve. The electrochemical active surface area (ECSA) is calculated as *C*_dl_/*C*_ds_, where *C*_s_ is 40 μF cm^−2^.

A 5 wt% Nafion solution was diluted 10 times to obtain a 0.5 wt% solution, which was then subjected to 30 min of ultrasonication in a water bath. Subsequently, 15 mg of the catalyst sample was weighed and added to 5 mL of 0.5 wt% Nafion solution, followed by 1 h of ultrasonication in a water bath, resulting in a catalyst ink. Using a microsyringe, 10 μL of the ink was drop-cast onto a glassy carbon electrode with a diameter of 4 mm. The electrode was left undisturbed at room temperature for 1 hour to allow the formation of a film covering the electrode surface. The geometric surface area of the rotating disk electrode (RRDE) was calculated to be 0.1256 cm^2^. Linear sweep voltammetry (LSV) polarization curve measurements were performed using a rotating disk electrode (RRDE) at a rotation speed of 1600 rpm and a scan rate of 5 mV s^−1^. The potential of the reference electrode Ag/AgCl was converted to the reversible hydrogen electrode (RHE), and all obtained potential values were adjusted according to the established formula *E*_RHE_ = *E*_Ag/AgCl_ + 0.059 pH + 0.198.^19^

## Results and discussion

**Fig. 1:**
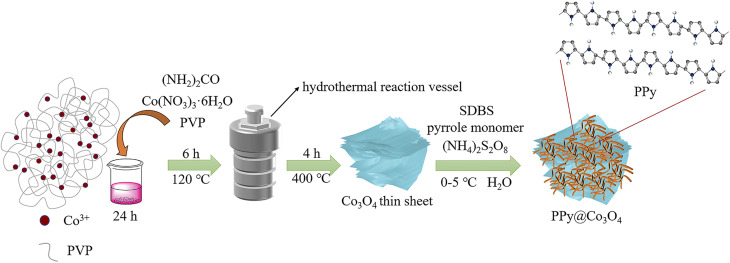
The preparation process of the PPy@Co_3_O_4_ electrocatalyst material.

As shown in Fig. 1, the multilayered Co_3_O_4_ nanosheets were synthesized using a simple hydrothermal method. Polyvinylpyrrolidone (PVP) was used as a soft template, and Co(NO_3_)_3_·6H_2_O was employed as the cobalt source to synthesize Co(OH)_2_ intermediate *via* urea. Subsequently, the Co_3_O_4_ nanosheets were obtained through a short calcination process. The synthesis of Co_3_O_4_ nanosheets is chiefly accomplished through the hydrothermal approach. Crucially, the use of PVP as a soft template plays a pivotal role, guiding growth along specific crystallographic planes while simultaneously suppressing other orientations, ultimately resulting in the formation of sheet-like architectures. To load PPy onto the surface of Co_3_O_4_ nanosheets, an *in situ* polymerization method was employed. The specific steps were as follows: SDBS surfactant, pyrrole monomer, and oxidant (NH_4_)_2_S_2_O_8_ were added into the Co_3_O_4_ nanosheets, and the *in situ* polymerization reaction was carried out at a controlled temperature range of 0–5 ^○^C. This resulted in the formation of a fibrous network structure of PPy attached to the surface of Co_3_O_4_ nanosheets, forming the PPy@Co_3_O_4_ composite material. The addition of SDBS surfactant facilitated the formation of stable micelle structures on the surface of Co_3_O_4_, ensuring the uniform dispersion of PPy nanoparticles on the Co_3_O_4_ surface and promoting the interaction between PPy and Co_3_O_4_.

**Fig. 2:**
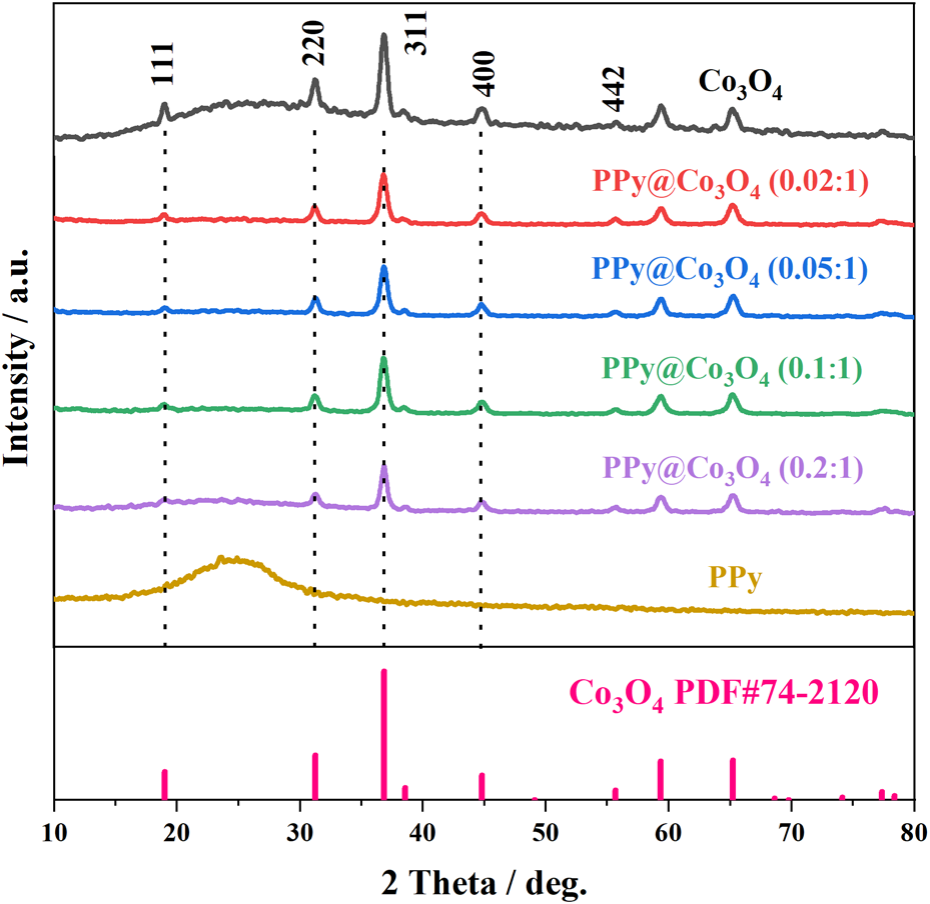
XRD patterns of the prepared Co_3_O_4_, PPy, and PPy@Co_3_O_4_ catalyst samples with different mass ratios.

Fig. 2 presents the XRD analysis of the prepared Co_3_O_4_, PPy, and PPy@Co_3_O_4_ catalyst samples with different mass ratios. The Co_3_O_4_ black powder exhibits distinct peaks at 18.9°, 31.3°, 36.8°, 44.8°, 55.6°, 59.3°, and 65.2°, which are in good agreement with the standard spectrum of Co_3_O_4_ (PDF#74-2120) and correspond to the (111), (220), (311), (400), (442), (511), and (440) crystal planes of Co_3_O_4_ product.^14^ This confirms the successful synthesis of the target product, Co_3_O_4_, through the hydrothermal decomposition of the precursor.^4^ Furthermore, the XRD patterns of the PPy@Co_3_O_4_ composite materials with different mass ratios also exhibit characteristic diffraction peaks of Co_3_O_4_, further confirming the presence of Co_3_O_4_ in the prepared catalyst samples.

**Fig. 3:**
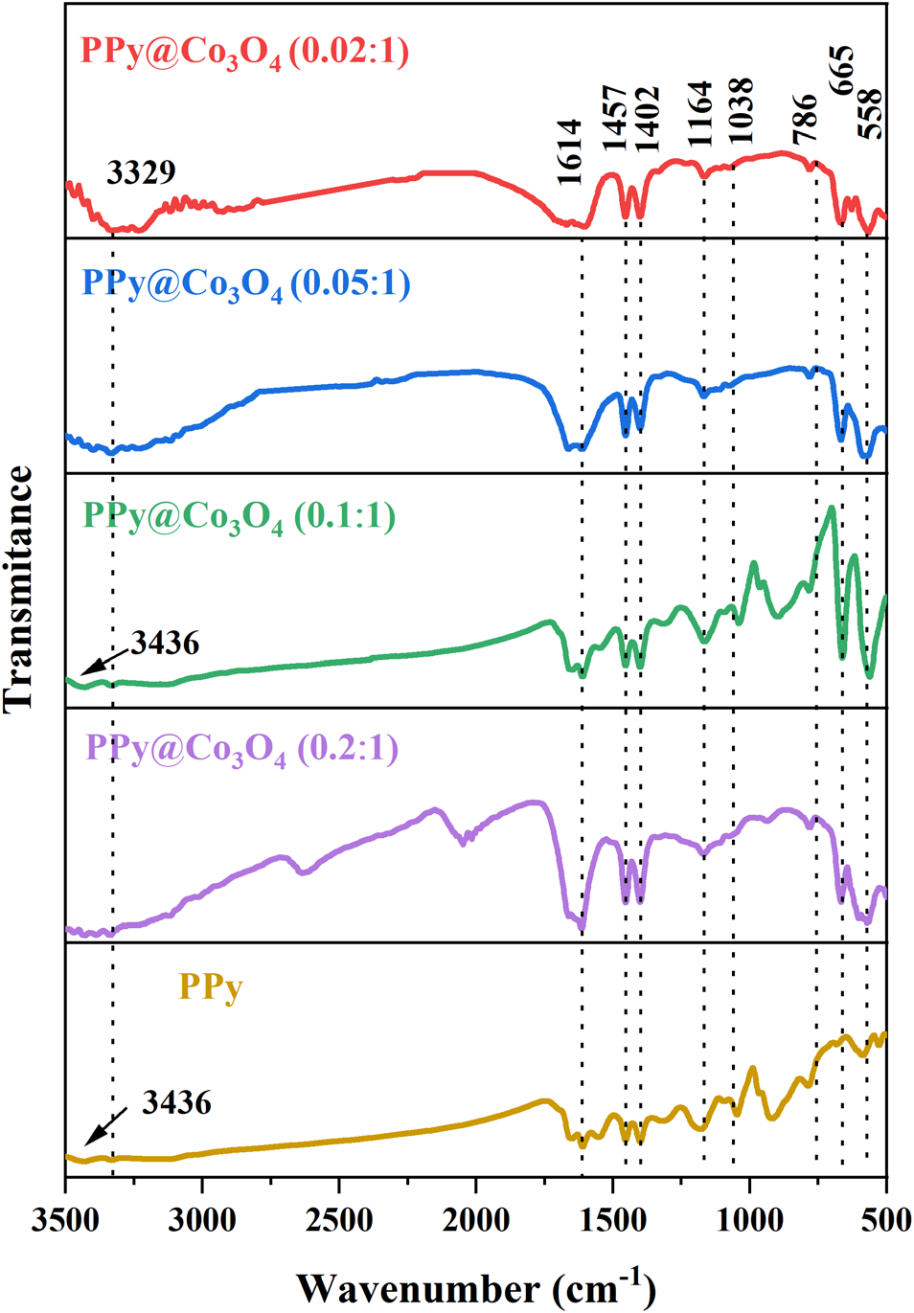
FT-IR spectra of PPy and PPy@Co_3_O_4_ catalysts with varying mass ratios.

To demonstrate the structure and composition of the prepared PPy@Co_3_O_4_ catalyst material, Fourier-transform infrared spectroscopy (FT-IR) analysis was conducted. As shown in Fig. 3, the spectrum analysis revealed two characteristic peaks at 3436 cm^−1^ and 3329 cm^−1^, which are attributed to the N–H stretching vibrations of aromatic amines.^20^ The peak at 1164 cm^−1^ corresponds to the stretching vibration of C–N bonds, while the absorption peak at 1038 cm^−1^ is attributed to the C–H vibration mode in the pyrrole ring.^21^ The peak observed at 1614 cm^−1^ corresponds to the stretching vibration of C=C bonds, and the peak at 558 cm^−1^ is attributed to the C–H vibration.^22^ These spectral peaks further confirm the presence of PPy in the prepared composite catalyst material. Additionally, a peak at 665 cm^−1^ corresponding to the Co–O bond^23^ was observed, indicating the presence of Co_3_O_4_ in the prepared composite catalyst material. Based on the analysis of the FT-IR characteristic peaks and the previous XRD test results, we can conclude that the prepared catalyst material is a PPy@Co_3_O_4_ composite material.

**Fig. 4:**
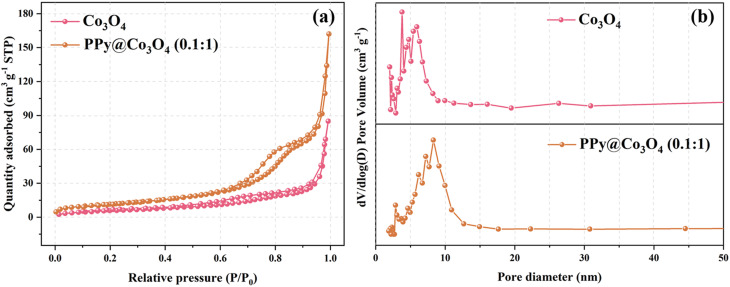
(a) N_2_ adsorption–desorption isotherms and (b) corresponding BJH pore size distribution curves for Co_3_O_4_ and PPy@Co_3_O_4_.

Based on the results of the N_2_ adsorption–desorption isotherm and pore size distribution curve, we observed that within the relative pressure range of 0 to 1.0, the catalyst samples displayed typical characteristics of a Type IV isotherm. As clearly depicted in Fig. 4(a), the BET surface area of the Co_3_O_4_ sample was measured to be 21.58 m^2^ g^−1^, whereas that of the PPy@Co_3_O_4_ (0.1 : 1) was notably higher at 41.83 m^2^ g^−1^. Fig. 4(b) provides a detailed representation of the pore size distribution of these samples: the average pore diameter of Co_3_O_4_ was approximately 13.26 nm, while that of PPy@Co_3_O_4_ (0.1 : 1) was slightly larger, around 13.88 nm. Notably, both materials predominantly exhibit a mesoporous structure. In a comprehensive comparison, PPy@Co_3_O_4_ (0.1 : 1) not only possesses a larger surface area but also a richer mesoporous structure, offering it numerous active sites, which are anticipated to significantly enhance its catalytic performance.^24–26^

**Fig. 5:**
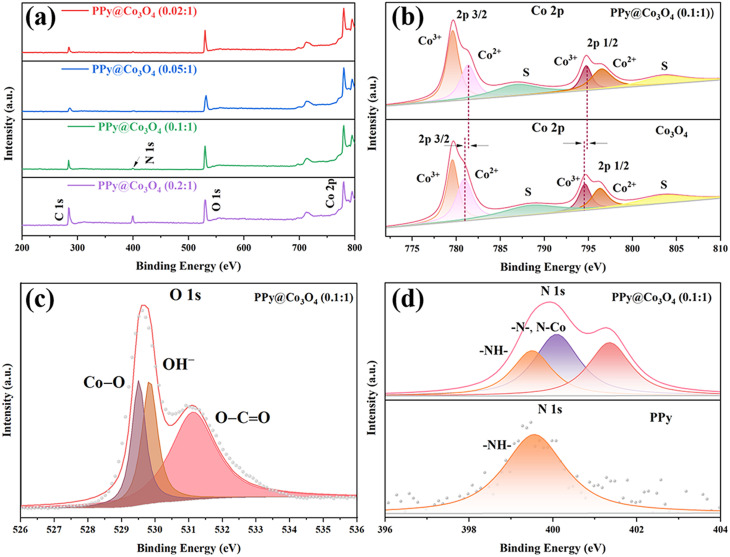
(a) XPS survey spectrum of PPy@Co_3_O_4_ composites. High-resolution XPS spectra of (b) Co 2p, (c) O 1s, and (d) N 1s for PPy@Co_3_O_4_ (0.1 : 1).

Based on XPS analysis, we have confirmed the chemical states of the PPy@Co_3_O_4_ catalyst surface. In the spectrum shown in Fig. 5(a), the presence of Co, N, O, and C elements is observed. As shown in Fig. 5(b), the Co 2p spectrum can be fitted with two spin–orbit doublets and two satellites. Specifically, the binding energies at 779.5 and 794.6 eV can be attributed to Co^3+^, while the binding energies at 796.3 and 780.9 eV can be attributed to Co^2+^. The energy gap of approximately 15.5 eV between 796.3 eV and 779.5 eV is consistent with the characteristic of Co 2p 1/2 and Co 2p 3/2 orbitals in Co_3_O_4_,^27,28^ further indicating the coexistence of Co^2+^ and Co^3+^. Furthermore, the shift of peaks in the Co 2p region towards higher binding energy suggests strong interactions between Co_3_O_4_ and PPy, leading to electron transfer from PPy to Co_3_O_4_.^29^ In Fig. 5(c), further analysis of the main peak in the O 1s region reveals three peaks at 529.5, 530.0, and 531.2 eV. These peaks are associated with oxygen in Co_3_O_4_. Specifically, the peak at 529.5 eV is attributed to the presence of metal–oxygen bonds, the binding energy at 530.0 eV corresponds to the presence of OH^−^, and the binding energy at 531.2 eV corresponds to low-coordinated oxygen ions.^30^

In the N 1s spectrum of PPy@Co_3_O_4_ (0.1 : 1) shown in Fig. 5(d) and S1(a),† the peak located at 399.2 eV matches with the N element in PPy, indicating a substantial presence of –NH– groups. The conjugated structure of PPy supports delocalized electrons, which exhibit electrostatic interactions with metal ions during the polymerization process.^31^ The peak at 400.6 eV corresponds to the C=N bond in PPy^4,32^ and the peak at 399.8 eV provides evidence for the formation of a Co–N bond, further substantiating the potential for electron transfer between PPy and Co_3_O_4_. Additionally, as observed in Fig. S1(b),† the C 1s region of PPy@Co_3_O_4_ catalysts in high-resolution XPS can be divided into four peaks. The peaks at 284.4, 285.2, 286.5, and 289.1 eV correspond to C–C, C–N, C–O, and C=O/C=N bonds, respectively.^20,33^ These results contribute to further characterizing the chemical composition and bonding states of the PPy@Co_3_O_4_ composite materials.

**Fig. 6:**
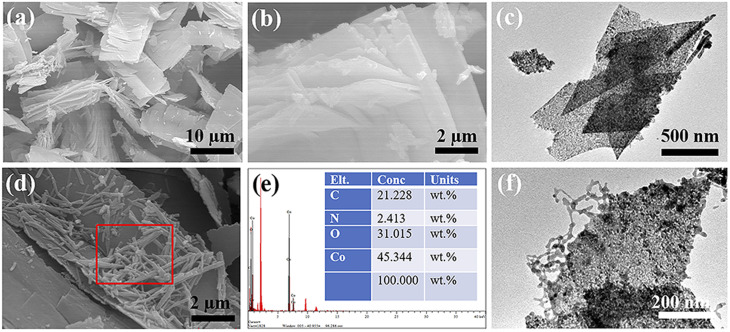
SEM images of Co_3_O_4_ at different magnifications: (a) low magnification and (b) high magnification. (c) TEM image of Co_3_O_4_. Characterization of PPy@Co_3_O_4_ catalyst: (d) SEM image, (e) EDS analysis, and (f) TEM image.

According to Fig. 6(a) and (b), the Co_3_O_4_ prepared using PVP as a template exhibits a multi-layered sheet-like structure. This is likely the result of the gradual growth of cobalt nitrate and urea precursor on the surface of the PVP template.^34^ With prolonged reaction time, the particle mass of the cobalt nitrate and urea precursor product gradually increases, leading to the gradual aggregation of initially independent nanocrystals through weak intermolecular interactions. After high-temperature calcination, they transform into a multi-layered Co_3_O_4_ nanosheet structure. This conclusion is further supported by the TEM results shown in Fig. 6(c), which confirms the prepared Co_3_O_4_ nanosheet structure. Fig. 6(d) displays the SEM image of the prepared PPy@Co_3_O_4_, clearly showing the distribution of PPy nanofibers on the surface of Co_3_O_4_ nanosheets. The corresponding EDS analysis results (Fig. 6(e)) demonstrate the elemental composition and content of PPy@Co_3_O_4_. The TEM results of PPy@Co_3_O_4_ in Fig. 6(f) reveal tightly and uniformly anchored PPy nanofibers on the surface of Co_3_O_4_ nanosheets. The elemental mapping images of PPy@Co_3_O_4_ (Fig. S2†) further corroborate this observation.

**Fig. 7:**
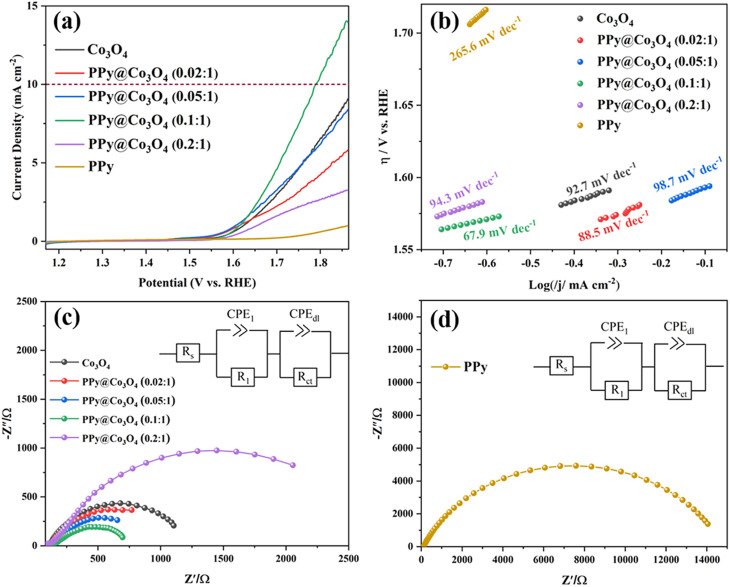
(a) OER polarization curves of catalysts obtained in 0.1 M KOH solution with a scan rate of 5 mV s^−1^. (b) Tafel plots. Electrochemical impedance spectra (EIS) for (c) Co_3_O_4_ and PPy@Co_3_O_4_ and (d) PPy.

By conducting LSV tests, as shown in Fig. 7(a), we compared the OER catalytic activity of Co_3_O_4_, PPy@Co_3_O_4_, and PPy. The results demonstrate that PPy exhibits inferior catalytic activity. In contrast, the optimized PPy@Co_3_O_4_ (0.1 : 1) catalyst exhibits the best catalytic performance, achieving a potential of 1.794 V *vs.* RHE at a current density of 10 mA cm^−2^, while the other PPy@Co_3_O_4_ and Co_3_O_4_ catalyst samples did not reach the potential for a current density of 10 mA cm^−2^. Compared to other catalysts, the optimized PPy@Co_3_O_4_ (0.1 : 1) catalyst demonstrates exceptional catalytic performance, achieving a potential of 1.794 V *vs.* RHE at a current density of 10 mA cm^−2^. Meanwhile, other PPy@Co_3_O_4_ and Co_3_O_4_ catalyst samples failed to attain this potential at the same current density. This indicates that as the content of PPy increases, its evolving network structure extensively covers the nanoplate surfaces, enhancing electron transport rates.

However, surpassing an optimal PPy content starts to negate these benefits, as active sites on the nanoplates could be permanently occluded by thick layers of PPy, hindering electron transfer. In short, polypyrrole plays a pivotal role in enhancing conductivity and acting as a conductive binder, but an excess of PPy can suppress the OER performance of the catalyst.^35–37^ Furthermore, by comparing the corresponding Tafel slopes shown in Fig. 7(b), it can be observed that PPy@Co_3_O_4_ (0.1 : 1) possesses the lowest slope value of 67.9 mV dec^−1^ among the catalyst samples, indicating higher catalytic activity and a faster OER reaction rate. Additionally, the catalytic activity of PPy@Co_3_O_4_ (0.1 : 1) is comparable to similar catalyst materials reported in the literature,^3,4,38,39^ as evident from the comparison in Table 1.

**Table 1:** Comparison of OER activity between PPy@Co_3_O_4_ composite catalyst and similar catalysts reported in the literature

Catalysts	Electrolyte (M)	OER onset potential (*E*/V *vs.* RHE)	Tafel slope (mV dec^−1^)	*E*_OER_ at 10 mA cm^−2^	Ref.
PPy@Co_3_O_4_ (0.02 : 1)	0.1	1.436	88.5		This work
PPy@Co_3_O_4_ (0.05 : 1)	0.1	1.433	98.7		This work
PPy@Co_3_O_4_ (0.1 : 1)	0.1	1.540	67.9	1.794	This work
PPy@Co_3_O_4_ (0.2 : 1)	0.1	1.544	94.3		This work
Co_3_O_4_/N-CNTs	1	1.37	40	1.55	3
Co_3_O_4_/PPy-120	1		57.7	1.45	4
Co_3_O_4_/PPy/RGO	0.1	1.298	105		38
IrO_2_	0.1	1.56	115	1.68	39

Fig. 7(c) and (d) present the electrochemical impedance spectroscopy (EIS) of PPy@Co_3_O_4_ at different mass ratios and pure PPy catalyst, respectively. The Nyquist plot is fitted based on an equivalent circuit model, as depicted in the inset. In this model, *R*_s_ represents the solution resistance; *R*_1_ corresponds to the electron transfer resistance from the catalyst to the electrode; and *R*_ct_ denotes the charge transfer resistance at the interface. Typically, a smaller *R*_ct_ value suggests a faster kinetic response of the catalyst. From the fitting data, it's observed that PPy@Co_3_O_4_ (0.1 : 1) has the lowest *R*_ct_ value of 630 Ω. In comparison, PPy@Co_3_O_4_ (0.02 : 1) and PPy@Co_3_O_4_ (0.05 : 1) exhibit *R*_ct_ values of 950 Ω and 1029 Ω, respectively. On the other hand, PPy@Co_3_O_4_ (0.2 : 1) displays a relatively higher impedance. This indicates that PPy@Co_3_O_4_ at a 0.1 : 1 mass ratio demonstrates the best charge transfer efficiency and enhanced catalytic activity.

Additionally, as shown in Fig. 7(d), the impedance of the pure PPy nanofibers is notably higher than that of PPy@Co_3_O_4_ composite catalysts at any given ratio. This further confirms that an appropriate amount of PPy can facilitate electron transport between PPy and Co_3_O_4_. This synergistic effect leads to a reduced charge transfer resistance at the interface between the electrolyte and the electrode, enhancing the overall charge transport performance and decreasing interfacial resistance.

To better elucidate the electrocatalytic activity of PPy@Co_3_O_4_, cyclic voltammetry (CV) tests were conducted on all the prepared PPy@CoCo_3_O_4_ composite materials at varying scan rates (as illustrated in Fig. S3 and S4†). From the CV curves depicted in Fig. S3,† the double-layer capacitance (*C*_dl_) values for the catalysts were calculated as follows: PPy@Co_3_O_4_ (0.02 : 1) 1.80 mF cm^−2^, PPy@Co_3_O_4_ (0.05 : 1) 4.35 mF cm^−2^, PPy@Co_3_O_4_ (0.1 : 1) 6.07 mF cm^−2^, and PPy@Co_3_O_4_ (0.2 : 1) 1.77 mF cm^−2^. Correspondingly, their electrochemical surface areas (ECSA) were 45.00 cm^2^ cm^−2^, 108.75 cm^2^ cm^−2^, 151.75 cm^2^ cm^−2^, and 44.25 cm^2^ cm^−2^, respectively. Notably, PPy@Co_3_O_4_ (0.1 : 1) exhibited the largest ECSA. ECSA is a pivotal metric in evaluating the performance of electrocatalysts. A larger ECSA often implies that more active sites are exposed to the reaction medium, frequently leading to enhanced OER catalytic activity. Additionally, an increased ECSA provides more pathways and channels for the efficient transport of electrolytes, intermediates, and products, thereby further optimizing catalytic performance.

**Fig. 8:**
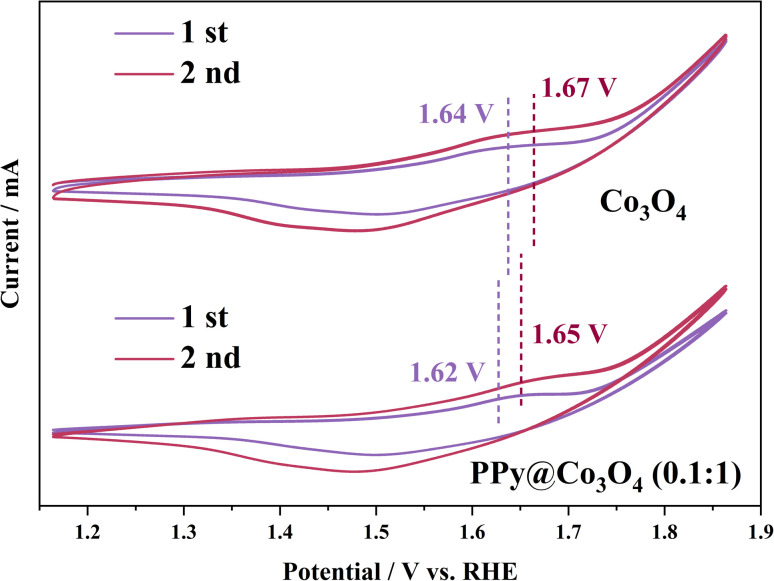
Pseudocapacitive behavior at first and second cycles during CV test for PPy@Co_3_O_4_ (0.1 : 1).

The pre-oxidation of the electrocatalyst plays a pivotal role in evaluating the active species through CV activation. CV activation promotes the transition from Co^2+^ to Co^3+^, highlighting the crucial step for efficient OER activity.^1,40^ As observed from Fig. S4(a)–(d),† oxidation peaks are evident at potentials of 1.6–1.7 V. With an increasing scan rate, the capacitive charge also rises, indicating an irreversible reaction on the electrocatalyst surface due to polarization. However, Fig. S4(e)† shows that an excessive content of polypyrrole impedes electron transfer, resulting in a less distinct polarization effect. As depicted in Fig. 8, the second cycle displays a more significant capacitive charge than the first cycle, suggesting that polarization occurred on the electrocatalyst surface, leading to enhanced structural stability. Moreover, for the PPy@Co_3_O_4_ (0.1 : 1) sample, the oxidation peak in the first cycle appears at 1.62 V and shifts to 1.65 V in the second cycle.

For Co_3_O_4_, oxidation peaks are observed at 1.64 V (first cycle) and 1.67 V (second cycle). This demonstrates that PPy can facilitate the pre-oxidation of Co^2+^, accelerating electron transfer.

**Fig. 9:**
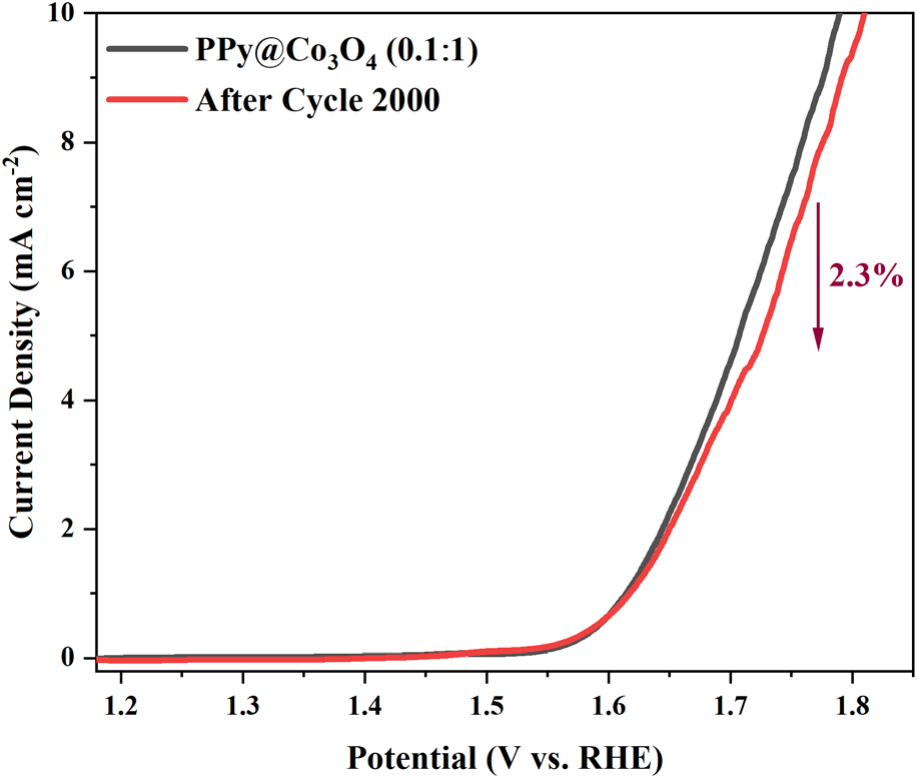
OER stability test of PPy@Co_3_O_4_ (0.1 : 1).

Based on the above analysis, it is evident that the composite catalyst structure, consisting of multi-layered Co_3_O_4_ nanosheets and attached PPy nanofibers, offers advantages such as increased effective reaction interface, abundant active sites, excellent charge transfer performance, and facilitated reactant diffusion. These characteristics collectively contribute to the enhanced OER catalytic activity of the catalyst.^16,41,42^ To evaluate the practical application potential of the PPy@Co_3_O_4_ (0.1 : 1) catalyst, we conducted cyclic stability testing with 2000 cycles of CV measurements, as shown in Fig. 9. The results demonstrated excellent stability performance, with a minimal decay rate of only 2.3% observed at the end of the test.

**Fig. 10:**
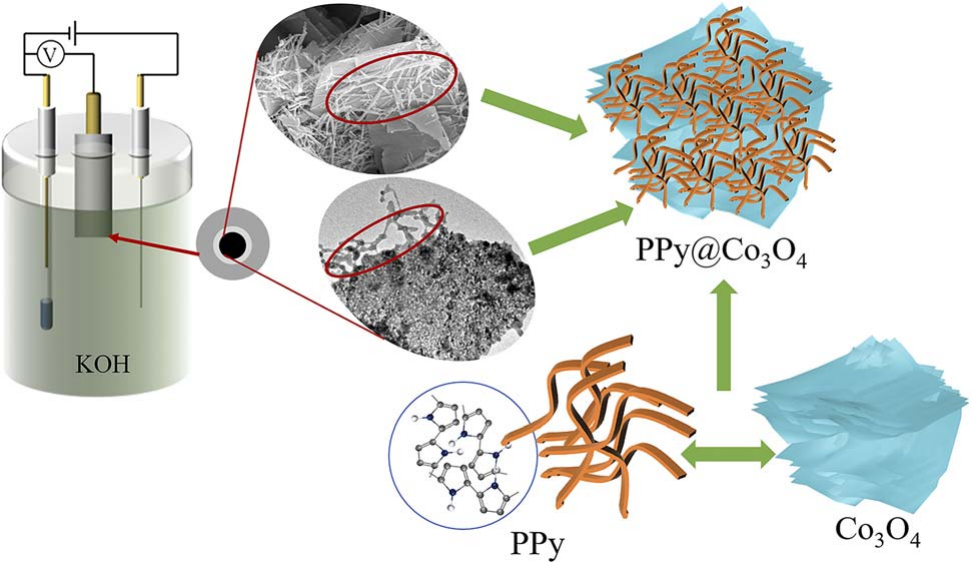
Mechanism diagram of the PPy@Co_3_O_4_ (0.1 : 1) catalyst.

Fig. 10 illustrates the mechanism diagram of the PPy@Co_3_O_4_ (0.1 : 1) catalyst. The multilayered thin flake-like Co_3_O_4_ provides a substantial surface area, while the attached PPy nanofibers further expand the reaction interface, creating abundant active sites for catalytic reactions. This unique composite structure significantly enhances the catalyst-electrolyte contact area, synergistically promoting catalytic efficiency. Moreover, the PPy nanofibers serve as a protective layer for the multilayered Co_3_O_4_ structure. They effectively shield Co_3_O_4_ from direct contact with the KOH electrolyte, reducing corrosion and dissolution of Co_3_O_4_, thus enhancing catalyst stability and maintaining long-term catalytic activity. Additionally, the flexible and tunable nature of PPy nanofibers mitigates volume changes and stress accumulation. The distinctive microstructural features of the PPy@Co_3_O_4_ (0.1 : 1) catalyst enable sustained high catalytic activity during prolonged use.

## Conclusions

In summary, we successfully synthesized a composite catalyst, PPy@Co_3_O_4_, consisting of multilayered thin-film Co_3_O_4_ with PPy nanofibers attached on the surface. The catalytic performance and stability of PPy@Co_3_O_4_ in OER were evaluated. Comparative analysis of Co_3_O_4_, PPy, and different mass ratios of PPy@Co_3_O_4_ catalyst samples revealed that PPy@Co_3_O_4_ (0.1 : 1) exhibited the highest OER catalytic activity. It demonstrated a lower overpotential at a current density of 10 mA cm^−2^, indicating a faster reaction rate in the OER. Tafel slope analysis further confirmed the rapid kinetic response of the PPy@Co_3_O_4_ (0.1 : 1) catalyst. Moreover, the CV stability test demonstrated excellent stability performance of the PPy@Co_3_O_4_ (0.1 : 1) catalyst with only a 2.3% decay after 2000 cycles. The unique structure of multilayered thin-film Co_3_O_4_ and attached PPy nanofibers provided numerous active sites, enhanced charge transfer properties, and a protective layer, effectively promoting the OER while minimizing catalyst corrosion and dissolution. These findings offer new insights and strategies for the development of efficient and stable OER catalysts with potential applications in energy conversion and storage.

## Conflicts of interest

There are no conflicts to declare.

## Supplementary Material

RA-013-D3RA05936G-s001
