# Supplementary material for: OER catalytic performance of a composite catalyst comprising multi-layer thin flake Co3O4 and PPy nanofibers
Source: RSC Adv. 2023 Oct 31;13(45):32045–53. doi: 10.1039/d3ra05936g (PMC10617464; doi:10.1039/d3ra05936g)
Supplement: RA-013-D3RA05936G-s001 [file RA-013-D3RA05936G-s001.pdf]

Electronic Supplementary Material (ESI) for RSC Advances.
This journal is © The Royal Society of Chemistry 2023

Supporting information

Composite Catalyst of Multi-Layer Thin Flake Co_3O_4 and PPy Nanofibers: Outstanding OER Catalytic Performance

Honglin Ai, Liquan Fan, Yuwei Wang*, Ziteng Wang, Juan Zhao, Haiming Zhang, Meiye Jiao, Boyu Lv, Xianxin Han*

College of Materials Science and Engineering, Heilongjiang Provincial Key Laboratory of Polymeric Composite Materials, Qiqihar University, No.42, Wenhua Street, Qiqihar 161006, PR China

*E-mail: Liquan_Fan@163.com; wyw032378@163.com

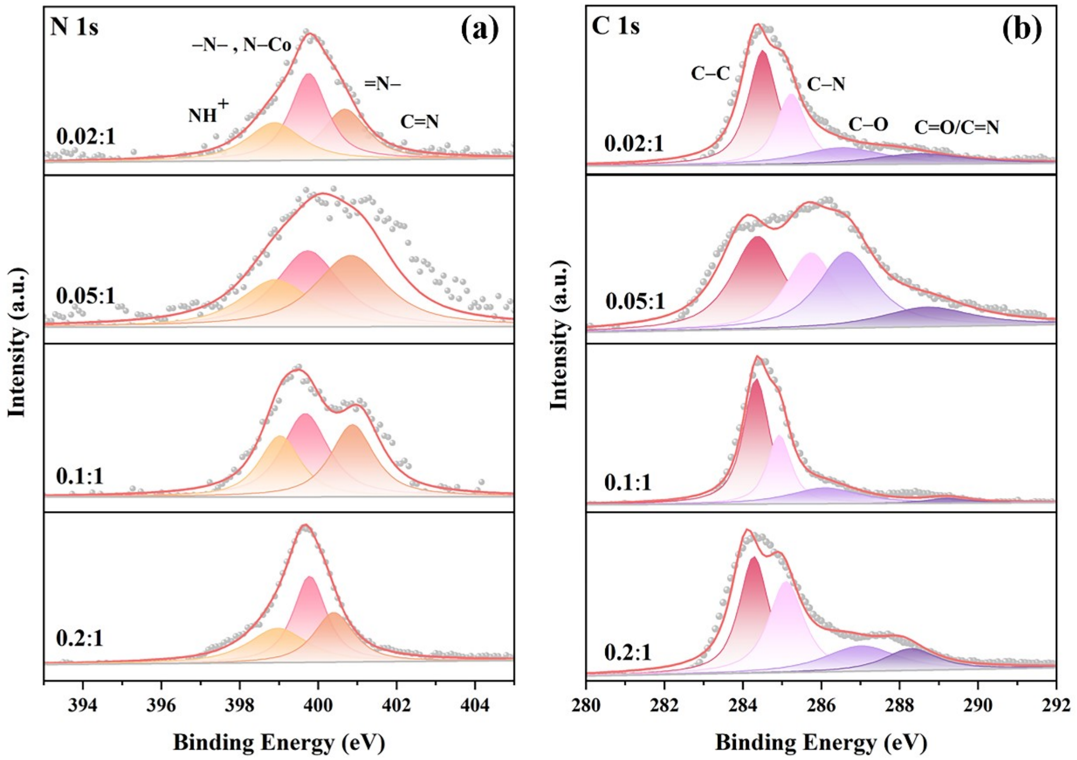

Fig. S1 High resolution XPS spectra of (a) N 1s and (b) C 1s for PPy@Co_3O_4 catalysts.

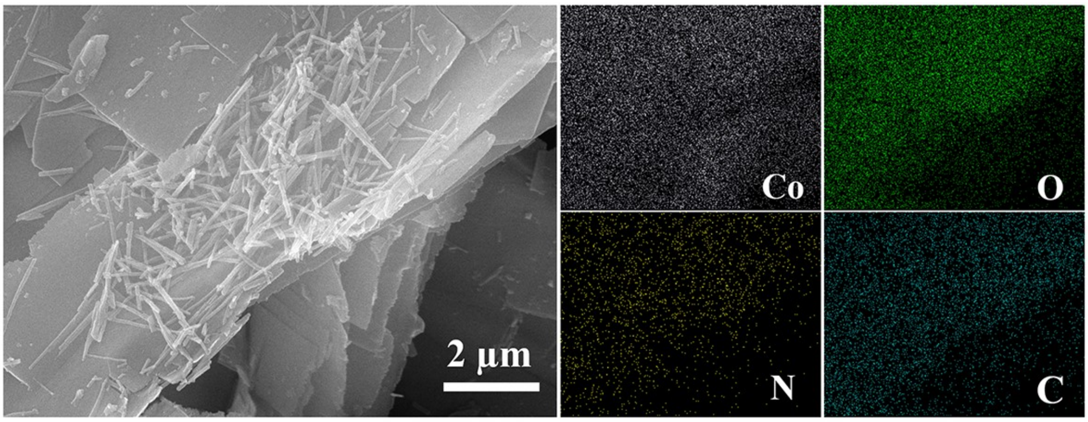

Fig. S2 SEM and corresponding elemental mapping of PPy@Co_3O_4 composite, revealing the elemental distributions of Co, O, N, and C.

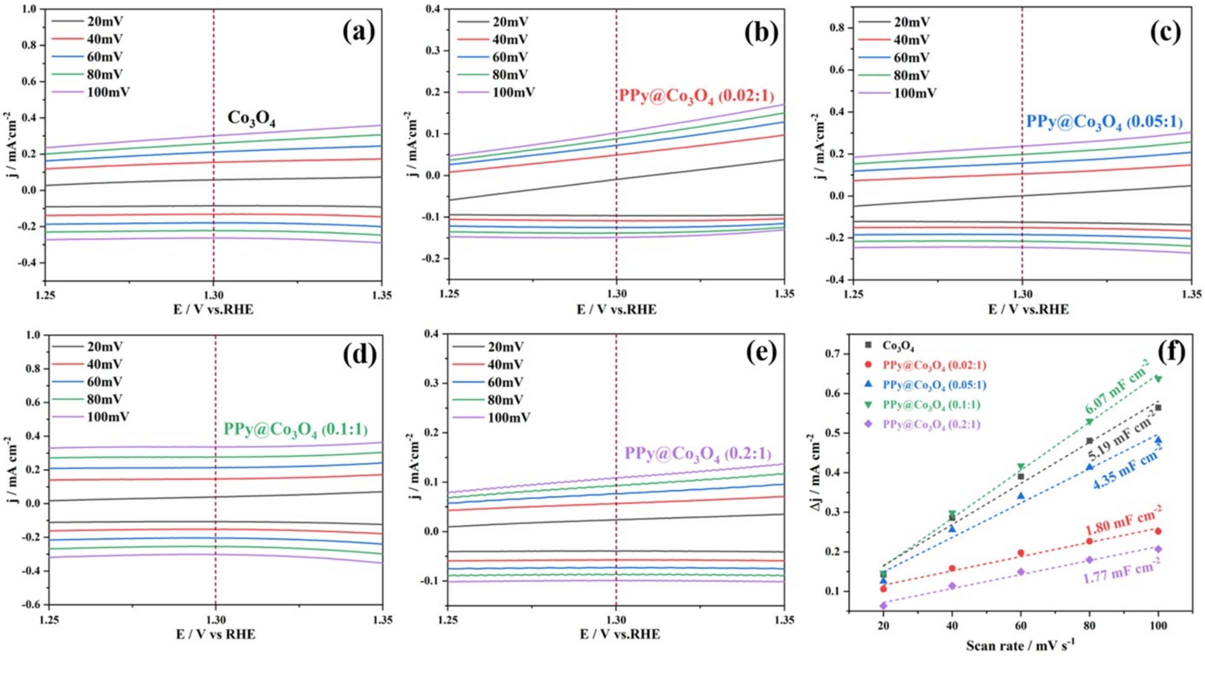

Fig. S3 (a)-(e) CV curves of composites obtained with different scan rates (20~100 mV s^{-1}) between a potential range of 1.25~1.35 V, (f) Relation curves between current density and scanning speed at 1.3 V

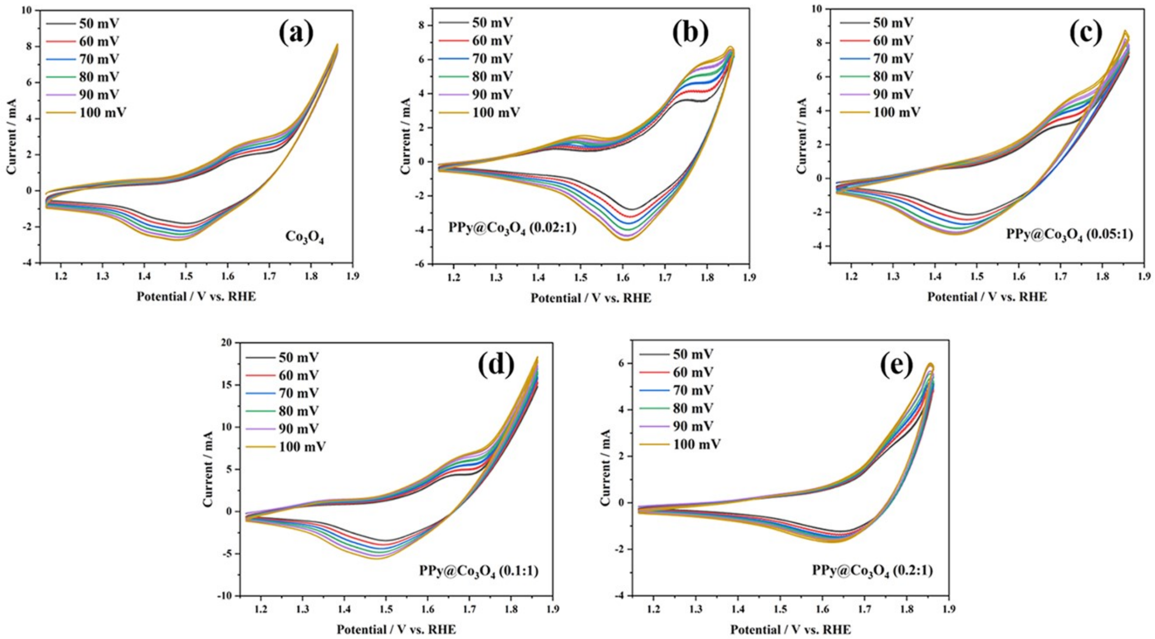

Fig. S4 CV curves of Co_3O_4 and PPy@Co_3O_4 composites obtained with different scan rates
